# Range Expansion Technology for Ring MEMS Gyroscopes Based on Drive Voltage Modulation

**DOI:** 10.3390/mi15121419

**Published:** 2024-11-26

**Authors:** Ke Cui, Li Liu, Daren An, Xinyu Wang, Qi An, Zengyu Lei, Zhiwei Kou, Huiliang Cao

**Affiliations:** 1 School of Instrument and Electronics, North University of China, Taiyuan 030051, China; 2 Mechanized Infantry Reconnaissance Department, The Army Infantry Academy of PLA, Shijiazhuang 050227, China; 3 College of Electric Power, Inner Mongolia University of Technology, Hohhot 010051, China; 4 Chongqing Institute of Microelectronics and Microsystems, Beijing Institute of Technology, Chongqing 401332, China

**Keywords:** MEMS ring gyroscope, range expansion, drive control loop, detecting open-loop, nonlinear, in-circuit debugging

## Abstract

This paper proposes a method to control the sensitivity of a ring MEMS gyroscope by adjusting the driving control voltage via MEMS. The aim is to explore the relationship between the range of the ring MEMS gyroscope and the driving control voltage, establishing a mathematical model that correlates driving control voltage with sensitivity. By applying different driving voltages to the same gyroscope, the study evaluates the performance and range of the gyroscope. Experimental results demonstrate that lower driving voltages increase the gyroscope’s range. At a driving voltage of 10.85 V, the gyroscope achieves a range of ±200°/s, a minimum resolution of 0.019°/s, and a nonlinearity of 22.37 ppm. At 1.46 V, the gyroscope range expands to ±1000°/s, with a minimum resolution of 0.05138°/s and a nonlinearity of 60.73 ppm. As the measurement range increased fivefold, the degradation in gyroscope performance was significantly less than the scale of range expansion. Compared to the gain in modulation detection circuitry, gyroscope performance was optimized across the entire operational range.

## 1. Introduction

With the rapid development of technology, micro-electromechanical systems (MEMS) technology has been widely applied in the fields of navigation, autonomous driving, and aerospace. As a high-precision and high-sensitivity inertial sensor, the ring MEMS gyroscope has received increasing attention due to its miniaturization and low power consumption [1,2]. As an important branch of integrated circuits, MEMS sensors are often limited in their range due to process and physical requirements. The detectable voltage range often limits the gyroscope’s dynamic range. After the design of the MEMS gyroscope is completed, the range modulation methods are effectively limited, which affects its performance under different measurement conditions [3,4].

Therefore, it is necessary to explore the range expansion and quantization technology of the control drive voltage based on the ring MEMS gyroscope. This allows the output range of the MEMS gyroscope to be autonomously adjusted online according to the usage scenario. At the same time, advanced circuit design and algorithms are combined to achieve low noise and high signal-to-noise ratio measurement results, ensuring the stability and reliability of the gyroscope in various working environments [5]. This innovative exploration provides new ideas for the future development of MEMS gyroscopes and lays a solid foundation for applications in related fields.

## 2. MEMS Ring Gyroscope Head Based on S-Shaped Elastic Beam

### 2.1. MEMS Ring Gyroscope Structure

**Figure 1:**
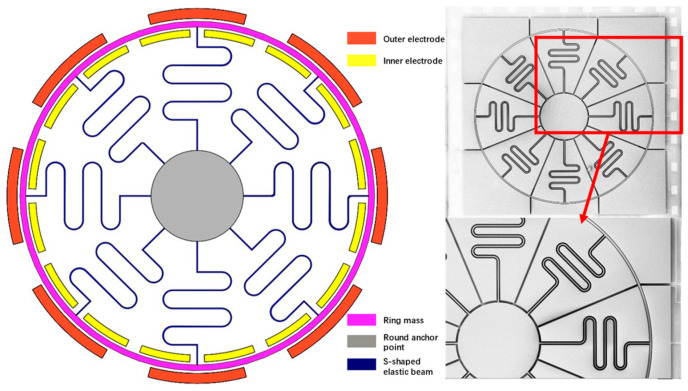
S-shaped ring gyroscope structure schematic diagram.

The MEMS ring gyroscope structure described in this article adopts a novel design scheme based on S-shaped beams, as shown in Figure 1. The entire structure consists of a central circular anchor point, a ring-shaped mass ring, eight sets of S-shaped beams, and inner and outer groups of eight electrodes. This structural design not only provides high flexibility and good mechanical stability but also effectively enhances the sensitivity and anti-interference capability of the gyroscope. The introduction of S-shaped beams ensures uniform stress distribution while maintaining excellent elastic recovery performance under high-stress conditions, significantly reducing the impact of nonlinear effects [6].

The MEMS ring gyroscope structure consists of a central circular anchor point, a ring-shaped mass ring, S-shaped beams, and inner and outer sets of eight electrodes. The central anchor point is the supporting part of the entire structure, and by fixing this anchor point, the balance of resonant motion of the ring-shaped mass ring under external excitation can be ensured. The design and material selection of the anchor point are crucial, as it must have sufficient mechanical strength to withstand impact and vibration during the high-frequency resonant motion of the ring-shaped mass ring while maintaining structural stability.

The ring-shaped mass ring is the core part of this design. It is connected to the S-shaped beams and is responsible for carrying the motion mass of the entire MEMS gyroscope. The displacement of the mass ring directly reflects the gyroscope’s response to external angular velocity input, determining the sensitivity and range of the MEMS gyroscope. By accurately designing the geometric dimensions and materials of the mass ring, the resonance frequency and detection performance of the gyroscope can be optimized [7].

The eight sets of S-shaped beams connecting the ring-shaped mass ring and the central anchor point are essential structures for the motion of the MEMS gyroscope. The shape and structure of the S-shaped beams offer unique advantages, playing a critical role in elastic recovery during the gyroscope’s resonant motion. When external angular velocity acts on the gyroscope, the S-shaped beams absorb and transfer mechanical energy through elastic deformation [8]. Compared to traditional straight beams, S-shaped beams distribute stress more evenly, reducing local stress concentration effects and minimizing nonlinear effects during high-voltage driving. The excellent recovery characteristics exhibited by S-shaped beams under high-stress conditions also effectively improve the long-term stability of the MEMS gyroscope [9].

### 2.2. Performance Testing of Gyroscope Head

The inner and outer groups of eight electrodes are key components for driving and detecting the MEMS gyroscope. The arrangement of the inner and outer electrodes generates electrostatic forces to drive the resonant motion of the ring-shaped mass ring while also detecting the displacement signals of the gyroscope. Electrostatic driving excites the vibration of the ring-shaped mass ring by applying voltage, and the capacitance changes between the inner and outer electrodes reflect the displacement of the mass ring. The design of the eight groups of electrodes enables multi-directional mechanical coupling, making the detection of angular velocity by the MEMS gyroscope more accurate [10].

**Figure 2:**
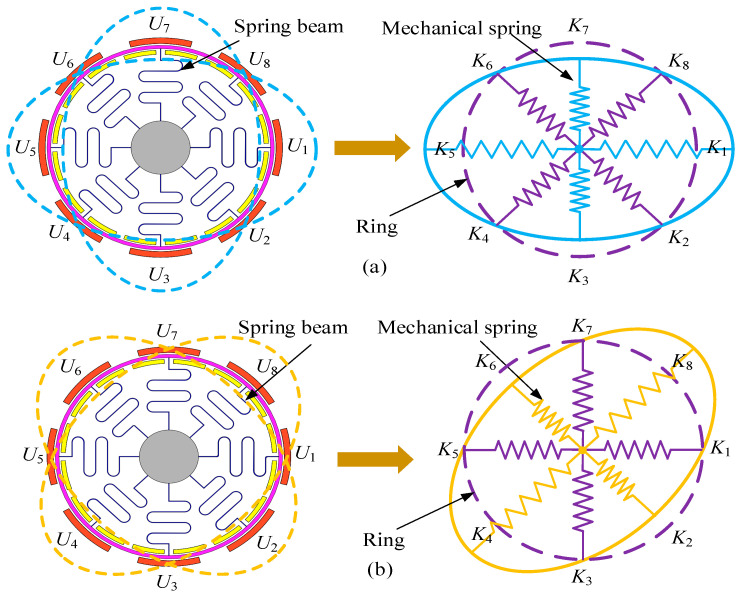
Diagram of the Drive Mode and Sense Mode of a Ring Gyroscope. (**a**) Drive mode. (**b**) Detection mode.

The working mode of the gyroscope head used in this test is shown in Figure 2. The performance parameters of the ring MEMS gyroscope mainly include the drive frequency, detection frequency, and quality factor, $Q_{x}=\frac{m_{x}\omega_{x}}{c_{x}}$ = 1200, $Q_{y}=\frac{m_{y}\omega_{y}}{c_{y}}$ = 1200 of the MEMS ring gyroscope head. The drive frequency ($F_{d}$) is 8424.6 Hz, which is the resonant frequency of the MEMS gyroscope under electrostatic drive. At a drive frequency of 8424.6 Hz, the gyroscope structure can resonate with maximum displacement, ensuring optimal detection sensitivity [11]. The detection frequency ($F_{s}$) is 8438.9 Hz, which is related to the gyroscope’s detection mode. At this detection frequency, the MEMS gyroscope can effectively respond to external angular velocity inputs. The detection frequency is very close to the driving frequency. This indicates that the design can achieve high-precision angular velocity detection and is suitable for high-precision measurement requirements such as inertial navigation [12].

**Figure 3:**
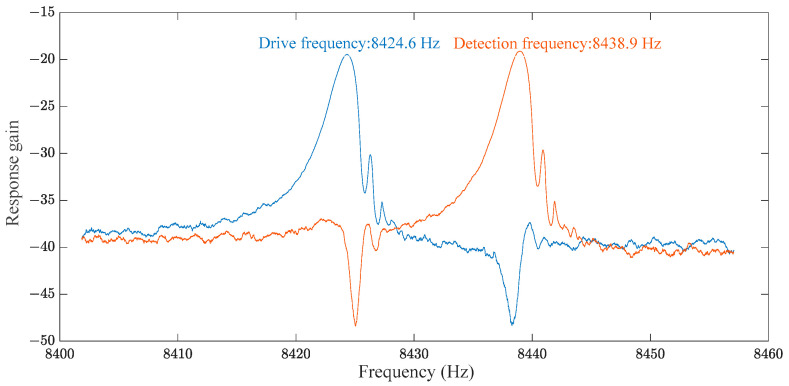
Scanning Results of MEMS Ring Gyroscope.

The quality factor is a critical parameter for measuring energy loss in the resonant motion of the MEMS gyroscope. A higher (Q) value indicates lower energy loss in the gyroscope’s resonant mode, resulting in more stable and enduring motion. The gyroscope head used in this test is shown in Figure 2. A higher quality factor indicates that the MEMS gyroscope has lower energy loss and can maintain a high mechanical quality factor during resonant motion, which is essential for improving detection accuracy and the long-term stability of the system [3,13]. The frequency sweep result of the MEMS gyroscope head used this time is shown in Figure 3.

### 2.3. Derivation of the Kinematic Equations for a Ring MEMS Gyroscope

The dynamic equation of the MEMS gyroscope structure resonator is given as:

(1) $$\left[\begin{array}{cc} m_{x} & 0\\ 0 & m_{y} \end{array}\right]\left[\begin{array}{c} \ddot{x}\\ {\ddot{y}}_{1} \end{array}\right]+\left[\begin{array}{cc} \frac{\omega_{x2}m_{x}}{Q_{x2}} & 0\\ 0 & \frac{\omega_{y1}m_{y1}}{Q_{y1}} \end{array}\right]\left[\begin{array}{c} \dot{x}\\ {\dot{y}}_{1} \end{array}\right]+\left[\begin{array}{cc} \omega_{x2}^{2}m_{x} & 0\\ 0 & \omega_{y1}^{2}m_{y1} \end{array}\right]\left[\begin{array}{l} x\\ y_{1}\\ y_{2} \end{array}\right]\left[\begin{array}{c} x\\ y_{1} \end{array}\right]=\left[\begin{array}{c} F_{dx}\sin\left(\omega_{d}t\right)\\ -2m_{c}\Omega_{z}\dot{x} \end{array}\right]$$

where *m_x_* and *m_y_* are the drive mode equivalent mass, sense mode equivalent mass and Coriolis mass; *x* and *y_1_* are drive mode displacement, sense in-phase mode displacement and sense anti-phase mode displacement, respectively; *Q_x_*_2_ and *Q_y_*_1_ are drive mode quality factor, sense in-phase mode quality factor and sense anti-phase mode quality factor, respectively; Ω*_z_* is angular rate input; *F_dx_* is drive mode stimulating magnitude; *ω_d_* is drive mode stimulating frequency; and *y*_1_ is total sense mode displacement, which gives us [6,14]:

(2) $$x(t)=\frac{F_{dx}\sin\left[\omega_{d}t-tg^{-1}\left(\frac{\omega_{x2}\omega_{d}}{Q_{x2}\left(\omega_{x2}^{2}-\omega_{d}^{2}\right)}\right)\right]}{m_{x}\sqrt{{\left(\omega_{x2}^{2}-\omega_{d}^{2}\right)}^{2}+\omega_{x2}^{2}\omega_{d}^{2}/Q_{x2}^{2}}}\overset{\omega_{d}=\omega_{x2}}{\to }x(t)=\frac{F_{dx}Q_{x2}}{m_{x}\omega_{d}^{2}}\cos(\omega_{d}t)=A_{x}\cos(\omega_{d}t)$$

(3) $$\begin{array}{l} y_{1}(t)=\frac{-2\Omega_{z}\omega_{d}F_{dx}\sin\left[\omega_{d}t-tg^{-1}\left(\frac{\omega_{x2}\omega_{d}}{Q_{x2}\left(\omega_{x2}^{2}-\omega_{d}^{2}\right)}\right)+\frac{\pi }{2}-tg^{-1}\left(\frac{\omega_{y1}\omega_{d}}{Q_{y1}\left(\omega_{y1}^{2}-\omega_{d}^{2}\right)}\right)\right]}{m_{x}\sqrt{{\left(\omega_{x2}^{2}-\omega_{d}^{2}\right)}^{2}+\omega_{x2}^{2}\omega_{d}^{2}/Q_{x2}^{2}}\sqrt{{\left(\omega_{y1}^{2}-\omega_{d}^{2}\right)}^{2}+\omega_{y1}^{2}\omega_{d}^{2}/Q_{y1}^{2}}}\\ \overset{\omega_{d}=\omega_{x2}}{\to }y_{1}(t)=\frac{-2\Omega_{z}F_{dx}Q_{x2}\sin(\omega_{d}t)}{m_{x}\omega_{d}\sqrt{{\left(\omega_{y1}^{2}-\omega_{d}^{2}\right)}^{2}+\omega_{y1}^{2}\omega_{d}^{2}/Q_{y1}^{2}}}=A_{y1}\sin(\omega_{d}t) \end{array}$$

So, the mechanical sensitivity of the MEMS ring gyroscope structure can be expressed as:

(4) $$S_{me}=\frac{A_{y1}}{\Omega_{z}}\approx \frac{-F_{dx}Q_{x2}}{m_{x}\omega_{d}^{2}}\left(\frac{1}{\omega_{y1}-\omega_{x2}}\right)=-A_{x}\left(\frac{1}{\Delta \omega_{1}}\right)$$

The drive force *F_dx_* generated by electrostatic force can be expressed as:

(5) $$F_{dx}=2\frac{\partial C_{d}(D+x)}{\partial x}V_{dc}V_{ac}=2\frac{A}{{\left(D+x\right)}^{2}}V_{dc}V_{ac}$$

Among these, C_d_ is a capacitor formed by the driving comb on one side. By inputting Equations (5) to (1), we can obtain:

(6) $$\ddot{x}+\frac{\omega_{d}}{Q_{x2}}\dot{x}+\omega_{d}^{2}x=2\frac{S}{{\left(D+x\right)}^{2}}V_{\mathrm{dc}}V_{dI}(t)\dot{x}$$

(7) $${\dot{V}}_{dI}(t)=K_{p}(V_{\mathrm{com}}-V_{dACA})\;+K_{i}\int (V_{\mathrm{com}}-V_{dACA})\;dt$$

**Figure 4:**
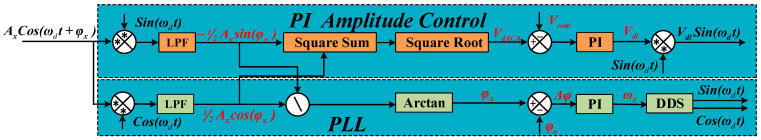
MEMS gyroscope drive mode closed-loop system.

As shown in Figure 4, $K_{f}$ is the system gain of the gyroscope drive loop. $\alpha_{d}$ is the gain coefficient of the low-pass filter in the measurement and control system. $\lambda_{d}$ is the angular frequency coefficient of the low-pass filter in the measurement and control system [15].

(8) $${\dot{V}}_{dACA}=\left|K_{f}\dot{x}\right|\alpha_{d}-\lambda_{d}V_{dACA}(t)\;$$

The drive mode speed and acceleration can be expressed as:

(9) $$\dot{x}=-A_{x}(t)\omega_{d}\sin(\omega_{d}t+\varphi_{x})$$

(10) $$\ddot{x}=-{\dot{A}}_{x}\omega_{d}\sin(\omega_{d}t+\varphi_{x})-A_{x}(t)\omega_{d}^{2}\cos(\omega_{d}t+\varphi_{x})$$

Substitute (2), (9) and (10) into (6) then obtain:

(11) $${\dot{A}}_{x}=2\frac{S}{{\left(D+A_{x}(t)\right)}^{2}}V_{\mathrm{dc}}V_{dI}(t)A_{x}(t)-\frac{\omega_{d}A_{x}(t)}{Q_{x2}}$$

Then, applying the average method, consider the average value in one period (T = 2π/ω_d_) of (7), (8) and (11), which can be obtained as:

(12) $${\dot{\bar{V}}}_{dI}(t)=K_{p}(V_{\mathrm{com}}-{\bar{V}}_{dACA})\;+K_{i}\int (V_{\mathrm{com}}-{\bar{V}}_{dACA})\;dt$$

(13) $${\dot{\bar{V}}}_{dACA}=\frac{2}{\pi }{\;A\;¯}_{x}\omega_{d}\alpha_{d}\left|K_{f}\right|-\lambda_{d}{\bar{V}}_{dACA}\;$$

(14) $${\dot{\bar{A}}}_{x}=2\frac{S}{{\left(D+{\bar{A}}_{x}(t)\right)}^{2}}V_{\mathrm{dc}}{\bar{V}}_{dI}(t){\bar{A}}_{x}(t)-\frac{\omega_{d}{\bar{A}}_{x}(t)}{Q_{x2}}\;$$

Let the right side of (12), (13), (14) equal zero, then get:

(15) $${\bar{V}}_{dACA0}=V_{\mathrm{com}}\;$$

(16) $${\;A\;¯}_{x0}=\frac{\pi \lambda_{d}}{2\omega_{d}\alpha_{d}\left|K_{f}\right|}\;V_{\mathrm{com}}\;$$

(17) $${\bar{V}}_{dI0}=\frac{\partial x\omega_{d}\left(D+x\right)}{2\partial C_{d}Q_{x2}\left|K_{f}\right|V_{\mathrm{dc}}}\;$$

**Figure 5:**
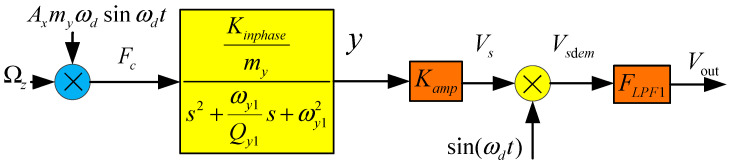
MEMS gyroscope sense mode opened-loop system.

In Figure 5, K_amp_ is the second differential amplifier; F_LPF1_ is the second order low pass filter, K_inphase_ is the displace-voltage transform parameters of the sensing in-phase and anti-phase modes, and the following equations can be obtained:

(18) $$\left\{\begin{array}{l} F_{c}\left(t\right)=2\Omega_{z}\left(t\right)m_{y}A_{x}\omega_{d}\sin\left(\omega_{d}t\right)\\ G_{sV/F}=\left(\frac{\frac{K_{inphase}}{m_{y}}}{s^{2}+\frac{\omega_{y1}}{Q_{y1}}s+\omega_{y1}^{2}}\right)\\ V_{s}=K_{amp}G_{sV/F}\\ V_{s\mathrm{dem}}\left(t\right)=V_{s}\left(t\right)\sin(\omega_{d}t+\varphi_{d0})\\ V_{out}=V_{s\mathrm{dem}}F_{LPF1} \end{array}\right.$$

After a series of transforms and calculations, the relationship between V_out_ and Ωz (the scale factor expression of the MEMS gyroscope sense closed-loop system) can be expressed as:

(19) $$\frac{V_{out}\left(s\right)}{\Omega_{z}(s)}=K_{amp}F_{LPF1}(s)m_{y}A_{x}\omega_{d}G_{sE}(s)$$

where G_sE_(s) is the gyroscope sense mode transform equation, which can be expressed as:

(20) $$G_{sE}=\left\{\frac{K_{inphase}(s^{2}+\frac{\omega_{y1}}{Q_{y1}}s+\omega_{y1}^{2}-\omega_{d}^{2})}{{\left(s^{2}+\frac{\omega_{y1}}{Q_{y1}}s+\omega_{y1}^{2}-\omega_{d}^{2}\right)}^{2}+{\left(2s\omega_{d}+\frac{\omega_{y1}}{Q_{y1}}\omega_{d}\right)}^{2}}\right.\left.\right\}$$

Then, substituting Equation (16) into Equation (20), the MEMS gyroscope scale factor can be obtained:

(21) $$\frac{V_{out}\left(s\right)}{\Omega_{z}(s)}=\frac{F_{LPFf}m_{y}\pi \lambda_{d}K_{amp}G_{sE}(s)V_{\mathrm{com}}}{2V_{dac}\left|K_{f}\right|\alpha_{d}}\;\;$$

So, the scale factor can be determined using K_amp,_ G_sE_(s), V_com_ is the control voltage that controls the motion amplitude of the driving mode. This parameter determines the motion amplitude of the driving mode and controls the sensitivity of the gyroscope’s detection mode. K_amp_ is the external gain at the back end of the gyroscope detection capacitor, which is a linear amplification. G_sE_(s) is the system transfer function of the gyroscope structure detection mode, determined by the gyroscope structure. After the gyroscope is produced, this transfer function is difficult to change.

## 3. MEMS Ring Gyroscope Range Expansion System

### 3.1. MEMS Gyroscope Detection Open-Loop Control System

**Figure 6:**
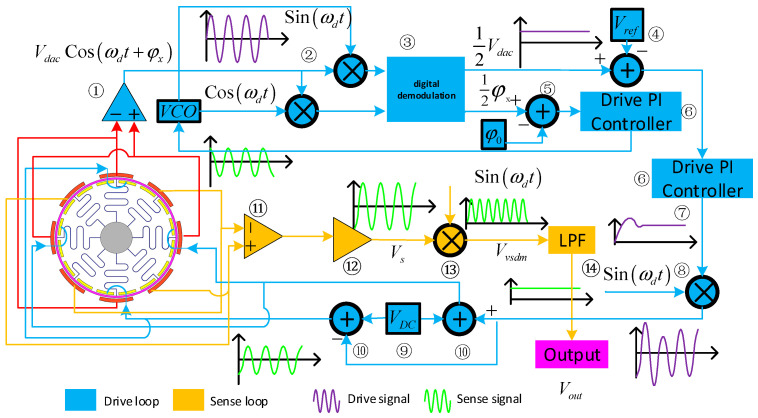
Open-Loop Measurement and Control System for Ring Gyroscope Detection.

The control and sensing system of the gyroscope is illustrated in Figure 6. In the drive circuit, the displacement (x(t)) of the drive frame is provided by the dynamic variation in capacitance of the differential drive detection capacitors. This is amplified by differential amplifier ➀, yielding the gyroscope motion signal ($V_{dac}Sin\left(\omega_{x}t\right)$).

Next, the readout signal is multiplied and demodulated with the reference signals ($Sin\left(\omega_{o}t\right)$) and ($Cos\left(\omega_{o}t\right)$) through step ➁, ensuring that the phase difference between the AC drive signal and the detection signal is maintained at 90°. Through this process, the amplitude and phase information of the gyroscope signal are extracted. The amplitude signal is (1/2 V_dac), and the phase signal is ($\varphi_{x}$). A digital resolver ➂ processes these signals, performing trigonometric operations and low-pass filtering. Subsequently, the (1/2 V_dac) signal (at stage ➃) is compared with the reference voltage ($V_{ref}$). Then, a PI controller ➅ generates a control signal, which is modulated with ($Cos\left(\omega_{o}t\right)$). This signal is superimposed via $V_{DC}$ ➈ and converted differentially (through step ➉) before being applied to the differential drive capacitor terminal of the gyroscope. At the frequency control loop, the phase signal is input into step ➄, where it is processed by a PI controller ➅. The resulting control signal is then sent to the VCO frequency generator, which produces the corresponding reference signals ($Sin\left(\omega_{o}t\right)$) and ($Cos\left(\omega_{o}t\right)$) [15].

### 3.2. Detecting the Relationship Between Open-Loop Range and Open-Loop Gain

Traditional range adjustment techniques for MEMS gyroscopes are typically limited by the range of the detection voltage amplitude in the circuit, especially when using circuits based on CMOS technology, where the voltage range is usually constrained to 0–3.3 V. This means that the system cannot significantly adjust the range or sensitivity by increasing the voltage and instead requires optimization through other means. To adapt to these limitations, traditional MEMS gyroscope range adjustment mainly relies on gain adjustment of the displacement signal in detection mode, particularly through gain adjustment in the C/V (capacitance-to-voltage) conversion circuit to achieve range adjustment [16,17].

During the detection process of the MEMS gyroscope, the displacement signal is usually manifested as a change in capacitance [18]. The C/V conversion circuit’s function is to convert these small capacitance changes into measurable voltage signals. However, due to the very weak displacement signal of MEMS sensors, it is difficult to effectively extract the signal using circuits within a standard voltage range alone. Therefore, gain adjustment has become a commonly used technique, obtaining system sensitivity and range through the gain output voltage of an operational amplifier [19,20].

The gain section of the C/V conversion circuit consists of an amplifying circuit with adjustable gain. This circuit typically includes an amplifier and a gain-adjustable resistor network. The main function of the amplifier is to preliminarily amplify the input signal, while the adjustable resistor network is used to flexibly control the gain magnitude. The gain size is primarily controlled by adjusting the voltage. In practical applications, the gain magnitude determines the amplitude of the circuit’s output signal, indirectly affecting the MEMS gyroscope’s range [21].

## 4. Range Online Modulation Technology Based on Driving Voltage

### 4.1. Tradeoff off About MEMS Range and Performance

In MEMS gyroscope design, the maximum range of the structure is typically determined based on the maximum application requirements. To meet the needs of different application scenarios, designers need to consider the trade-off between range and sensitivity in the structural design of the MEMS gyroscope. Generally, the larger the range, the worse the detection sensitivity [22]. This is because during the design process, the detection sensitivity of the structure is inversely related to the range. In other words, to adapt to a larger range, a portion of the sensitivity is often sacrificed in the design of the MEMS gyroscope. This balance between range and sensitivity is one of the key challenges in MEMS gyroscope design [23].

In practical applications, signal detection in MEMS gyroscopes primarily relies on the motion displacement signal of the structure. Although traditional gain adjustment techniques based on back-end signal processing can amplify the electrical signal output of the MEMS gyroscope, this amplification applies to all signals, including both the valid signal and noise [24]. This means that when the gain circuit amplifies the signal, both the valid signal and noise are amplified, and the gain circuit itself may introduce additional noise.

For example, in a gain amplification circuit, thermal noise and low-frequency noise from the amplifier and resistor network will interfere with the final output signal. Particularly when using a large range, due to the unchanged sensitivity of the MEMS gyroscope structure, the amplitude of the output signal becomes smaller as the resolution of the circuit decreases. This means that the proportion of random noise and external interference within the valid signal increases, ultimately leading to a degradation of system performance [4].

Further analysis reveals that when a smaller range is used, the sensitivity of the gyroscope structure remains constant, and the circuit gain is set higher to ensure that the output signal amplitude is sufficiently large [25]. Although the resolution of the gyroscope detection system matches the signal at the detection end, the noise level will also increase due to the influence of gain amplification. Therefore, system noise must be controlled within a certain range, usually requiring noise less than 100 nV/√ Hz to ensure the effectiveness and accuracy of the signal [26].

At smaller ranges, the overall performance of the MEMS gyroscope is usually better, as the proportion of random noise and external interference within the valid signal is lower, resulting in a higher signal-to-noise ratio and more stable output signals. However, as the range increases, performance gradually deteriorates. This is because the resolution of the circuit is fixed, and when the measurement range is large, the amplitude of the MEMS gyroscope’s output signal becomes smaller, making noise and interference impacts on the signal more pronounced.

Additionally, although range adjustment based on external circuits allows for sensitivity and range optimization within a certain range, this method does not fundamentally solve the problem of performance consistency. To meet broad range adjustment requirements, MEMS gyroscopes are generally designed with lower structural sensitivity. This approach ensures basic functionality over a larger range, but due to lower structural sensitivity, the performance of the MEMS gyroscope significantly declines at high ranges, particularly in terms of signal resolution and noise immunity.

Within this design framework, the MEMS gyroscope cannot ensure performance consistency over the entire range, especially at large ranges where system performance significantly degrades. This degradation is manifested not only in increased noise and reduced signal quality but may also prevent the MEMS gyroscope from meeting high-precision measurement requirements in certain applications. Therefore, in large-range applications, more complex signal processing and calibration techniques are required to compensate for the adverse effects caused by reduced sensitivity and increased noise.

### 4.2. The Relationship Between Gyroscope Driving Voltage and Sensitivity

Our team explored a technique based on controlling the range of the gyroscope by adjusting the drive voltage of the ring MEMS gyroscope, aiming to improve the performance consistency of the MEMS gyroscope across the full range. The core of this technique is to control the displacement of the MEMS gyroscope’s structural motion by adjusting the drive voltage, thereby changing its detection sensitivity and range to optimize system performance. Research has shown that there is a proportional relationship between the detection mode sensitivity of the gyroscope and the drive motion displacement of the MEMS structure, providing theoretical support for optimizing system performance through drive voltage adjustment.

**Figure 7:**
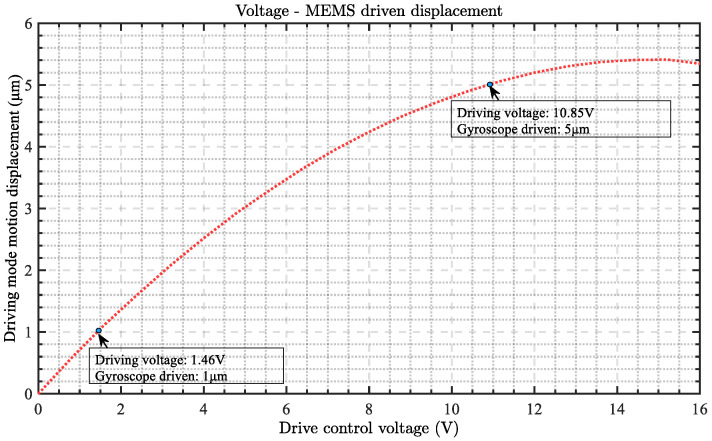
Relationship between driving voltage and MEMS gyroscope driving displacement.

Firstly, we test the relationship between the driving voltage (*V_com_*) and the vibration displacement of the MEMS gyroscope driving mode. As shown in Figure 7, the relationship between the driving voltage and the motion displacement of the gyroscope is not entirely linear. When the driving voltage is between 0 V and 12 V, the motion displacement of the gyroscope is positively correlated with the driving voltage, showing a good linear fit. The voltage drive in this stage can significantly change the motion displacement of the gyroscope, thereby altering its detection sensitivity to meet a wide range of requirements.

When the drive voltage exceeds 12 V, noticeable nonlinear effects start to appear in the system’s performance. At this point, the increase in the gyroscope’s motion displacement slows down with higher drive voltage, the displacement gain gradually decreases, and the correlation between voltage and displacement worsens. This nonlinear effect not only affects the system’s sensitivity adjustment but also negatively impacts the stability of the gyroscope’s motion. The main cause of this nonlinear effect is the increased stress in the MEMS structure under high voltage, particularly the stress concentration phenomenon in the S-shaped beam section of the ring mass structure, leading to deviations in structural displacement response.

Therefore, we explored the direct relationship between driving voltage and gyroscope sensitivity, and in this study, two voltage testing points were selected for testing. For the gyroscope, 1.46 V and 10.85 V are used as driving voltages. At this voltage, when the measurement and control systems are stable, the driving displacement of the gyroscope head is calculated as 5 μm and 1 μm, respectively. Both driving voltages can achieve stable operation in the gyro mirror driving mode. We will test the detection sensitivity of the gyroscope under two voltage control conditions and further explore the relationship between them.

**Figure 8:**
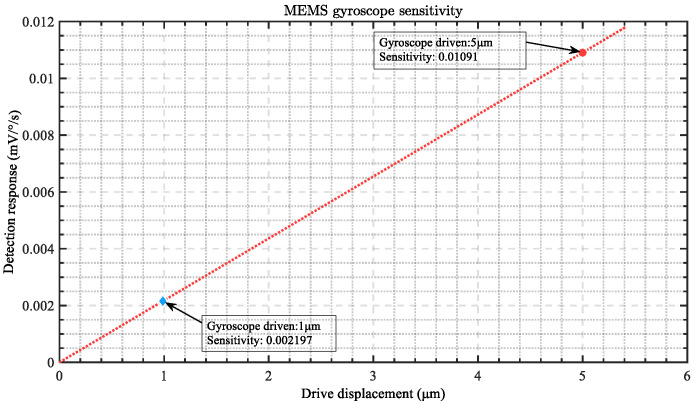
Relationship between driving displacement and gyroscope detection sensitivity.

According to Formula (21), the sensitivity of the gyroscope is related to V_com_*_,_* K_amp_ and G_Se_(s). Firstly, we analyze that in a mature system, the adjustment of the total gain of the detection circuit in the measurement and control system is always difficult and limited. Some of the drawbacks to this have been discussed earlier. For the gyroscope head, its internal parameters have already been determined during design, and the difficulty and cost of modification are high, which is not conducive to direct adjustment. The vibration displacement of the driving mode of the gyroscope is better directly adjusted. In the previous section, we tested the changes in vibration displacement of the gyroscope driving mode under multiple driving voltages. We substituted the displacement results of the test drive into Formula (21) for calculation, as shown in Figure 8. We selected two representative driving voltages for sensitivity testing, with calculated sensitivities of 0.002197 mV/°/s and 0.01091 mV/°/s.

## 5. Experiment and Test Analysis

### 5.1. Performance Testing of Gyroscope Under High Driving Voltage

The size of the experimental gyroscope measurement and control system is 50 mm × 40 mm × 40 mm, and the size of the gyroscope sensitive head is 32 mm × 30 mm × 10 mm. The power supply voltage of the experimental gyroscope measurement and control system is 5 V, the startup working current is 200 mA, and the working power consumption is 1 W.

**Figure 9:**
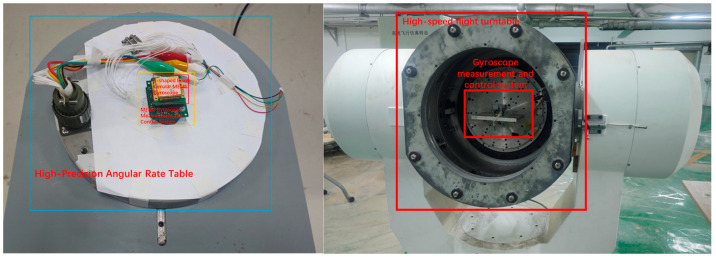
Schematic Diagram of Sensor Testing and Calibration System.

When the driving voltage of the gyroscope is maintained at 10.85V, the driving mode displacement of the gyroscope is 5 μ m, and the sensitivity ratio of the gyroscope detection mode is 0.01091 mV/°/s. After the MEMS gyroscope measurement and control system stabilizes, the team uses a high-precision angular velocity turntable to calibrate and test the gyroscope. The STZ-2A single axis position rate turntable with an accuracy of 0.001°/s and a testing range of up to 350°/s is used to support the calibration of angular rate sensors. We selected the following angular velocity values for calibration testing: ±300°/s, ±200°/s, ±100°/s, ±50°/s, ±20°/s, ±10°/s, ±5°/s, ±2°/s, ±1°/s, ±0.5°/s, ±0.2°/s, ±0.1°/s, ±0.05°/s, ±0.02°/s, ±0.01°/s, and ±0.005°/s. The installation method of the gyroscope measurement and control system is shown in Figure 9, which is installed at the center of the turntable to ensure accurate capture of angular velocity changes during the testing process and reduce the influence of external interference on the results.

**Figure 10:**
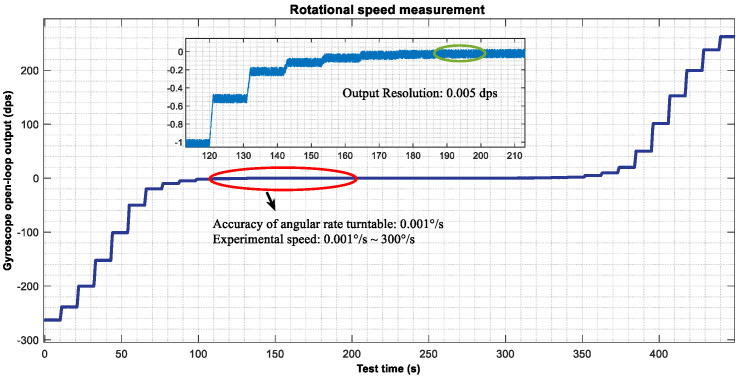
MEMS gyroscope angular velocity test.

The experimental results are shown in Figure 10. Under the control of 10.85 V driving voltage, the minimum output resolution is 0.005 dps. Under this driving voltage, the gyroscope measurement and control system maintains high resolution at low angular velocities, meeting the precise measurement requirements in small-scale applications. This feature gives MEMS gyroscopes significant advantages in high-precision, low-speed angular velocity measurement scenarios, particularly suitable for applications sensitive to small angular velocity changes, such as inertial navigation and attitude control.

**Figure 11:**
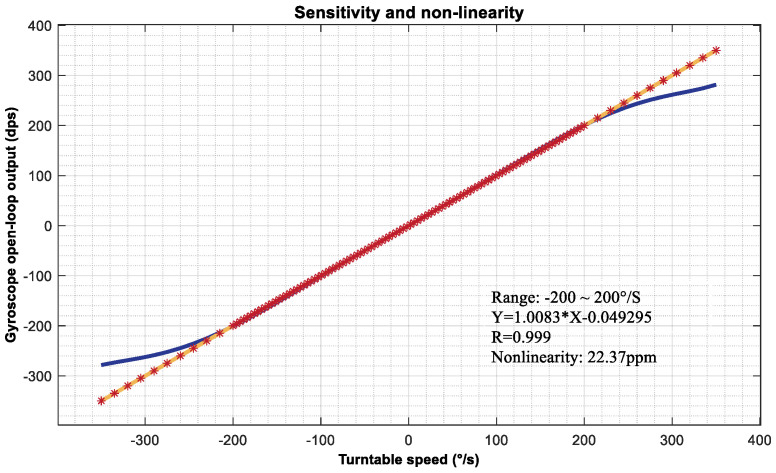
Nonlinear MEMS gyroscope.

The fitting results of the system output and input angular velocity are shown in Figure 11. Within the range of ±200°/s, the output of the gyroscope measurement and control system can be fitted as (Y=1.0083X−0.049295), where X is the input angular velocity and B is the static offset term. The nonlinear calculation formula is $\delta =\frac{\max\left(Y_{i}-\bar{Y}\right)}{Y_{Fs}}\times 100,000=22.37\;\mathrm{ppm}$. This indicates excellent performance within this range, capable of achieving high-precision linear output. Outside the range of ±200°/s, the system output exhibits extremely poor nonlinearity, so the current measurement and control system range is only ±200°/s.

Although gyroscope measurement and control systems exhibit high linearity and resolution in a small range, their performance is affected by coupled Coriolis forces in a larger angular velocity range. When the external input angular velocity exceeds a certain limit, the coupled Coriolis force within the MEMS ring structure begins to significantly affect the motion of the gyroscope, especially in the 45° detection mode, where the displacement of the ring structure exhibits linear degradation. Research has shown that this phenomenon is mainly due to nonlinearity in the annular mass structure, which is closely related to stress concentration in the S-shaped beam structure. Under high angular velocity input, the stress generated in the beam structure leads to a decrease in displacement gain, thereby affecting the operational stability and measurement accuracy of the gyroscope.

### 5.2. Comparison Testing

**Figure 12:**
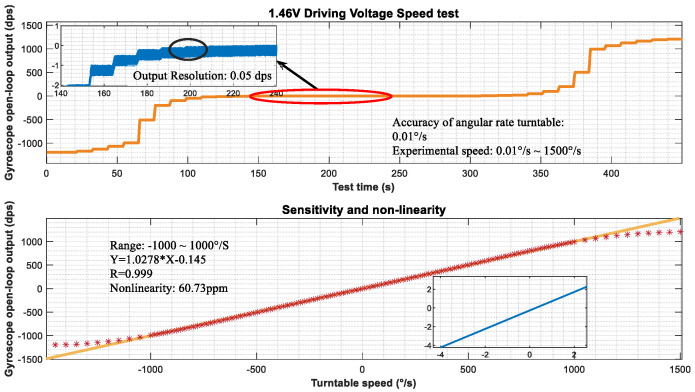
Range amplification test results.

As a comparative verification test, the driving voltage of the measurement and control system was adjusted to 1.46 V, and the vibration displacement of the gyroscope driving mode in the current state was 1 μm, with a sensitivity ratio of 0.002197 mV/°/s. The testing uses a SJT series three-axis precision angular motion turntable with an angular velocity control accuracy of 0.01°/s and an input range of ±1500 °/s, providing support for the calibration of angular velocity sensors. The installation diagram of the measurement and control system is shown in Figure 9. The following angular velocity values are selected for calibration testing: ±1500°/s, ±1000°/s, ±500°/s, ±200°/s, ±100°/s, ±50°/s, ±20°/s, ±10°/s, ±5°/s, ±2°/s, ±1°/s, ±0.5°/s, ±0.2°/s, ±0.1°/s, and ±0.05°/s. The installation method of the gyroscope measurement and control system is shown in Figure 12, which is installed at the center of the turntable. The experimental results are shown in Figure 12. Under the control of a driving voltage of 1.46 V, the minimum output resolution is 0.05 dps. Within the effective range of ±1000 °/s, the linear relationship of the gyroscope output can be expressed as Y=1.0278 X−0.145, with a minimum resolution of 0.05138°/s and a nonlinearity of 60.73 ppm. The gyroscope expanded using the range extension technology mentioned in this article does not change the system response time of the gyroscope itself, so the response time is the same as that of the open-loop detection loop of the gyroscope.

Comparing the results of two driving voltage tests, the lower driving voltage allows for a 500% increase in the measurable range, which is proportional to the amplitude range of the gyroscope driving mode vibration, and controls the degradation of gyroscope performance caused by noise. Adjusting the control voltage of the gyroscope driving mode can adjust the sensitivity of the gyroscope in real-time online to achieve the different ranges of the gyroscope. Without changing the gain factor of the detection circuit, digital compensation and fusion in the measurement and control system can adapt to any range within ±200°/s to ±1000°/s. When the vibration displacement of MEMS gyroscope structures decreases, the influence of external interference signals (such as vibration, temperature, etc.) on the gyroscope structure itself will be amplified, and the performance of the gyroscope measurement and control system will degrade significantly. It also puts forward higher requirements for the accuracy of the measurement and control system. Higher range and lower sensitivity of sensors under the same voltage conditions require higher resolution of analog-to-digital conversion circuits in measurement and control.

### 5.3. Model of Gyroscope Driving Voltage and Sensitivity

**Figure 13:**
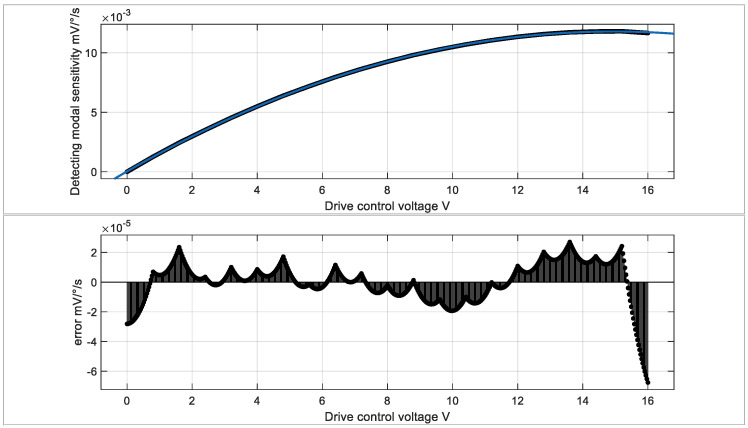
Sensitivity and driving voltage fitting and residual analysis.

Figure 13 further analyzes the direct relationship between driving voltage and gyroscope sensitivity. The upper figure shows that when the amplitude of the driving voltage changes, the detection sensitivity also changes accordingly. The lower figure shows the maximum residual of the fitting result. This sensitivity adjustment mechanism helps to precisely control MEMS ring gyroscopes. By adjusting the amplitude of the driving voltage, dynamic control of gyroscope sensitivity can be achieved to meet different application requirements.

Further experimental fitting analysis shows that within the drive voltage range of 0–15 V, the relationship between the drive voltage and the gyro sensitivity can be accurately fitted using the cubic polynomial equation: $y=6.862\;*\;10^{-9}x^{3}-5.291\;*\;10^{-5}x^{2}+0.001576\;*\;x+2.817\;*\;10^{-5}$. The fitting quality is as high as 0.9999, with system residuals not exceeding ($10^{-4}$). This fitting equation indicates a corresponding relationship between the drive voltage and sensitivity, providing a mathematical basis for the precise calibration and optimization of the system.

Based on this fitting formula, the gyroscope’s range can be tested by adjusting different drive control voltages. The range is controllably adjustable from ±200°/s to ±1000°/s. Across the full test range, the current sensitivity and range of the gyroscope can be well predicted through software calculation and compensation, indicating that the MEMS ring gyroscope exhibits good adaptability in a wide range of angular velocity measurement applications.

## 6. Conclusions

In practical applications, this adjustable range feature provides greater flexibility for different measurement scenarios. For example, under high-precision requirements, a lower range can be selected to enhance sensitivity, whereas for wide-range requirements, the measurement range can be expanded by reducing the drive voltage, ensuring measurement stability and accuracy. This strategy of optimizing range and sensitivity based on drive voltage adjustment, combined with the miniaturization and low power consumption characteristics of MEMS devices, provides a broad application prospect for vibrating ring gyroscopes in modern navigation, attitude control, and motion sensing fields. Additionally, this adjustable parameter design lays a foundation for future system integration and adaptive control.

## Data Availability

The data used to support the findings of this study are available from the corresponding author upon request.

## References

[1] Bryzek J. (1996). Impact of MEMS technology on society. Sens. Actuators A Phys..

[2] Ren X., Zhou X., Yu S., Wu X., Xiao D. (2021). Frequency-modulated MEMS gyroscopes: A review. IEEE Sensors J..

[3] Cao H., Liu Y., Kou Z., Zhang Y., Shao X., Gao J., Huang K., Shi Y., Tang J., Shen C. (2019). Design, fabrication and experiment of double U-beam MEMS vibration ring gyroscope. Micromachines.

[4] Cao H., Cai Q., Zhang Y., Shen C., Shi Y., Liu J. (2021). Design, fabrication, and experiment of a decoupled multi-frame vibration MEMS gyroscope. IEEE Sens. J..

[5] Cao H.-L., Li H.-S., Wang S.-R., Yang B., Huang L.-B. (2013). Temperature compensation of monitoring circuit for silicon MEMS gyroscope. Opt. Precis. Eng..

[6] Cui M., Huang Y., Wang W., Cao H. (2019). MEMS gyroscope temperature compensation based on drive mode vibration characteristic control. Micromachines.

[7] Sun J., Yu S., Zhang Y., Shi Y., Lu K., Xi X., Wu X., Xiao D. (2022). Characterization and compensation of detection electrode errors for whole-angle micro-shell resonator gyroscope. J. Micromachines. Syst..

[8] Gu H., Su W., Zhao B., Zhou H., Liu X. (2020). A design methodology of digital control system for MEMS gyroscope based on multi-objective parameter optimization. Micromachines.

[9] Cao H., Liu Y., Zhang Y., Shao X., Gao J., Huang K., Shi Y., Tang J., Shen C., Liu J. (2019). Design and experiment of dual-mass MEMS gyroscope sense closed system based on bipole compensation method. IEEE Access.

[10] Nguyen M.N., Ha N.S., Nguyen L.Q., Chu H.M., Vu H.N. (2017). Z-axis micromachined tuning fork gyroscope with low air damping. Micromachines.

[11] Kou Z., Liu J., Cao H., Han Z., Sun Y., Shi Y., Ren S., Zhang Y. (2018). Investigation, modeling, and experiment of an MEMS S-springs vibrating ring gyroscope. J. Micro/Nanolithogr. MEMS MOEMS.

[12] Miao T., Ou F., Xu Q., Hou Z., Wu X., Xiao D. (2018). A novel method of quadrature compensation in the butterfly resonator based on modal stiffness analysis. AIP Adv..

[13] Hou Z., Kuang Y., Ou F., Xu Q., Miao T., Xiao D., Wu X. (2021). A quadrature compensation method to improve the performance of the butterfly vibratory gyroscope. Sens. Actuators A Phys..

[14] Bu F., Guo S., Fan B., Wang Y. (2022). Effect of quadrature control mode on ZRO drift of MEMS gyroscope and online compensation method. Micromachines.

[15] Xu X., Liu X., Zhang Y. (2021). Design of a digital control system of disk gyroscope with orthogonal control circuit. Mod. Phys. Lett. B.

[16] Marano D., Cammarata A., Fichera G., Sinatra R., Prati D. (2017). Modeling of a three-axes MEMS gyroscope with feedforward PI quadrature compensation. Advances on Mechanics, Design Engineering and Manufacturing, Proceedings of the International Joint Conference on Mechanics, Design Engineering & Advanced Manufacturing (JCM 2016), 14–16 September 2016, Catania, Italy.

[17] Cao H., Li H., Kou Z., Shi Y., Tang J., Ma Z., Shen C., Liu J. (2016). Optimization and experimentation of dual-mass MEMS gyroscope quadrature error correction methods. Sensors.

[18] Cao H., Xue R., Cai Q., Gao J., Zhao R., Shi Y., Huang K., Shao X., Shen C. (2020). Design and experiment for dual-mass MEMS gyroscope sensing closed-loop system. IEEE Access.

[19] Antonello R., Oboe R., Prandi L., Caminada C., Biganzoli F. Open loop compensation of the quadrature error in MEMS vibrating gyroscopes. Proceedings of the 35th Annual Conference of IEEE Industrial Electronics.

[20] Cai Q., Zhao F., Kang Q., Luo Z., Hu D., Liu J., Cao H. (2021). A novel parallel processing model for noise reduction and temperature compensation of MEMS gyroscope. Micromachines.

[21] Han M., Zhang Q., Hao S., Li W. (2019). Parametric characteristics and bifurcation analysis of multi-degree-of-freedom micro gyroscope with drive stiffness nonlinearity. Micromachines.

[22] Cao H., Li H., Shao X., Liu Z., Kou Z., Shan Y., Shi Y., Shen C., Liu J. (2018). Sensing mode coupling analysis for dual-mass MEMS gyroscope and bandwidth expansion within wide-temperature range. Mech. Syst. Signal Process..

[23] Feng R., Wang J., Qiao W., Wang F., Zhou M., Shang X., Yu L., Zhou L., Guo S. (2021). Real-time built-in self-test of MEMS gyroscope based on quadrature error signal. Micromachines.

[24] Ding X., Ruan Z., Jia J., Huang L., Li H., Zhao L. (2021). In-run mode-matching of MEMS gyroscopes based on power symmetry of readout signal in sense mode. IEEE Sensors J..

[25] Bu F., Fan B., Xu D., Guo S., Zhao H. (2021). Bandwidth and noise analysis of high-Q MEMS gyroscope under force rebalance closedloop control. J. Micromech. Microeng..

[26] Xu P., Wei Z., Guo Z., Jia L., Han G., Si C., Ning J., Yang F. (2021). A real-time circuit phase delay correction system for MEMS vibratory gyroscopes. Micromachines.

